# Narrow-Banded UVB Affects the Stability of Secondary Plant Metabolites in Kale (*Brassica oleracea* var. *sabellica*) and Pea (*Pisum sativum*) Leaves Being Added to Lentil Flour Fortified Bread: A Novel Approach for Producing Functional Foods

**DOI:** 10.3390/foods8100427

**Published:** 2019-09-20

**Authors:** Rebecca Klopsch, Susanne Baldermann, Alexander Voss, Sascha Rohn, Monika Schreiner, Susanne Neugart

**Affiliations:** 1 Leibniz Institute of Vegetable and Ornamental Crops (IGZ) e.V., Theodor-Echtermeyer-Weg 1, 14979 Grossbeeren, Germany; Baldermann@igzev.de (S.B.); Schreiner@igzev.de (M.S.); 2 NutriAct – Competence Cluster Nutrition Research Berlin-Potsdam, Nuthetal, 14558, Germany; Alexander.Voss@igv-gmbh.de (A.V.); Rohn@chemie.uni-hamburg.de (S.R.); 3 Institute for Food Chemistry, Hamburg School of Food Science, Universität Hamburg, Grindelallee 117, 20146 Hamburg, Germany; 4 Germany Department of Food Chemistry, University of Potsdam, Institute of Nutritional Science, Arthur-Scheunert-Allee 114-116, 14558 Nuthetal, Germany; 5 Institute for Food and Environmental Research (ILU) e. V., Arthur-Scheunert-Allee 40-41, 14558 Nuthetal, Germany; 6 Division Quality and Sensory of Plant Products, Department of Crop Sciences, Georg-August-Universität Göttingen, Carl-Sprengel-Weg 1, 37075 Goettingen, Germany; susanne.neugart@uni-goettingen.de

**Keywords:** narrow-banded UVB, thermal processing, antioxidant activity, kale, pea, flavonoids, carotenoids

## Abstract

Young kale and pea leaves are rich in secondary plant metabolites (SPMs) whose profile can be affected by ultraviolet B (UVB) radiation. Carotenoids and flavonoids in kale and pea exposed to narrow-banded UVB, produced by innovative light-emitting diodes (LEDs), and subsequently used for breadmaking were investigated for the first time, thus combining two important strategies to increase the SPMs intake. Breads were also fortified with protein-rich lentil flour. Antioxidant activity in the ‘vegetable breads’ indicated health-promoting effects. Lentil flour increased the antioxidant activity in all of the ‘vegetable breads’. While carotenoids and chlorophylls showed a minor response to UVB treatment, kaempferol glycosides decreased in favor of increasing quercetin glycosides, especially in kale. Additionally, breadmaking caused major decreases in carotenoids and a conversion of chlorophyll to bioactive degradation products. In ‘kale breads’ and ‘pea breads’, 20% and 84% of flavonoid glycosides were recovered. Thus, kale and pea leaves seem to be suitable natural ingredients for producing innovative Functional Foods.

## 1. Introduction

To facilitate the consumer to counteract increasing diet-related illnesses by consuming a healthier diet, traditional foods can be modified and enhanced with natural health-promoting ingredients [1]. The key health benefit of these so-called Functional Foods is that they contain increased concentrations of bioactive compounds such as secondary plant metabolites (SPMs) [1]. SPMs represent a large group of bioactive compounds. Flavonoids and other SPMs, e.g., carotenoids and chlorophyll derivatives, are known for their antioxidant activity, which is linked to disease-preventing properties that are dependent on the chemical structure of the different metabolites [2,3,4]. Therefore, Shahidi and Ambigaipalan [5] proposed that the health-promoting properties of SPMs are more likely related to a diet with diversified antioxidant intake rather than the consumption of a single antioxidant. Thus, consuming vegetables, which naturally contain a multitude of antioxidant SPMs, might lead to better health-promoting effects compared to taking dietary supplements alone [5]. Besides a diet rich in SPMs, an increased protein intake is also recommended for elderly people (age > 65 years) to optimize their state of health and longevity [6]. Indeed, a high intake of plant protein has been associated with a lower risk for mortality caused by cardiovascular diseases compared to a high intake of animal protein [7]. Lentils are a traditional source of plant proteins, rich in flavonoids and other SPMs, and could therefore also possess health-promoting effects [8,9]. Thus, lentil flour seems to be a suitable ingredient for enhancing the protein content in Functional Foods. It should, however, be considered that proteins and polyphenols can interact in the food matrix and influence each other’s bioavailability [10,11]. When aiming to develop a Functional Food with increased SPMs and protein content, such interactions have to be taken into account. Several SPMs, such as carotenoids, chlorophylls, hydroxycinnamic acid derivatives, and flavonoids, are involved in plants response to UV (ultraviolet) radiation [12,13]. Low doses of UV radiation as suitable elicitors for enhancing SPMs to improve the nutritional quality of vegetables are the focus of numerous studies [14,15]. Within the spectrum of natural, solar UV radiation, the energy density and adverse biological efficiency (e.g., DNA damage) increases from 400 nm/UVA and is highest at 280 nm/UVB [16]. To date, the majority of studies have examined broad spectrum UV radiation with light bulbs that often peak at 310 nm, while including both UVA and UVB radiation [17]. However, the more recent emergence of light-emitting diodes (LED) technology enables researchers to study how the SPM profile can be optimized via UVB regarding its health-promoting potential [18]. Thus, the first objective of the present study was to examine the impact of pre-harvest, narrow-banded UVB treatment (290 nm) on carotenoids, chlorophylls, hydroxycinnamic acid derivatives, and flavonoid glycosides with the explicit aim of enhancing SPM production. In addition to pre-harvest treatments, post-harvest treatments, such as thermal processing, are also known to have a significant impact on the SPM profile [19,20]. Indeed, our own previous studies dealing with a fortification of bread with non-processed plant material revealed that the impact of breadmaking on SPMs is highly dependent on the chemical structure of the individual SPMs [21,22]. Consistent with other studies on the fate of SPMs during thermal processing [19,20], carotenoids and chlorophyll were found to decrease to a great extent during the breadmaking process. Moreover, bioactive chlorophyll degradation products were also observed during the breadmaking process and hydroxycinnamic acid derivatives as well as flavonoids were found to be relatively stable or even increase [21,22]. Kale, a leafy *Brassica* species, is known for its high concentrations of the carotenoid lutein, which has been suggested to delay the onset and progression of macular degeneration [23]. Kale also contains complex glycosylated and acylated kaempferol as well as quercetin glycosides [21,24]. In contrast, pea leaves contain less complex kaempferol and quercetin glycosides [22,25]. We aimed to facilitate an increased consumption of health-promoting SPMs, by combining two crucial topics in life science: elicitation of SPMs in planta with narrow-banded UVB, produced by innovative LEDs, and studying the impact of thermal processing on SPMs. Consequently, the objectives of this study were (1) to investigate whether SPMs can be triggered by exposure to pre-harvest, narrow-banded UVB radiation, and if so, (2) to assess, for the first time, whether these compounds exhibit a different behavior during the breadmaking process. As plant species with promising bioactive compound profiles, kale and pea were used to produce a Functional Food. Additionally, lentil flour was also added to increase the protein concentration. It is questionable if polyphenol–protein interactions affect the bioactive compounds in the ‘vegetable bread’. 

## 2. Materials and Methods

### 2.1. Chemical and Reagents

Ammoniac (NH_3_), ammonium acetate, boric acid, ferulic acid, hydrochloric acid (HCl), kaempferol-3-*O*-glucoside, methanol, natrium hydroxide (NaOH), quercetin-3-*O*-glucoside, sinapic acid, and *tert*-butyl methyl ether were obtained from Carl Roth GmbH + Co. KG (Karlsruhe, Germany). Acetic acid, 2-propanol, 2,2-diphenyl-1-picrylhydrazyl (DPPH), methylene chloride, potassium persulfate, and Trolox^®^ (6-hydroxyl-2,5,7,8-tetramethylchroman-2carbonic acid) were purchased from Merck KGaA (Darmstadt, Germany). 2,2′-Azinobis-(3-ethylbenzthiazoline-6sulfonic acid) (ABTS) was purchased from Roche Diagnostic GmbH (Mannheim, Germany) and sulfuric acid from Grüssing GmbH (Filsum, Germany). Acetonitrile was obtained from J.T. Baker (Fisher Scientific GmbH, Grießheim, Germany) and tetrahydrofurane was from VWR International GmbH (Darmstadt, Germany). The standards of β-carotene, chlorophyll a, chlorophyll b, lutein, and zeaxanthin were purchased from CaroteNature^®^ GmbH (Münsingen, Switzerland) and pheophytin a from LGC Standards GmbH (Wesel, Germany). 

### 2.2. Plant Material

For kale (*Brassica oleracea* var. *sabellica*) and pea (*Pisum sativum*), the cultivars *Redbor* (Volmary Digital GmbH, Münster, Germany) and *Blauwschokker Kapuzinererbse* (Sperli GmbH, Everswinkel, Germany) were used, respectively. Plants were grown in a climate chamber in trays without being separated (Supplemental Materials Figure S1). The temperature was 22 °C and 18 °C during day and night, respectively. Plants were watered *ad libitum* and no fertilizer was added. Until the 10th day, plants were illuminated with sun-simulating broad-banded light (including UVA and UVB) with halogen sodium metal halide lamps (Clean Ace™ R MT400DL/BH YE, EYE Lighting Europe Ltd, Middlesex, United Kingdom) with a light intensity of 500 µmol m^−2^ s^−1^ (UVB 0.0189 W/m², UVA 69.502 W/m²) and a 12/12 h photoperiod. To study the effects of pre-harvest, narrow-banded UVB light treatment, kale and pea plants were illuminated for 2 h each day from the 10th to the 13th day of plant growth with narrow-banded UVB LEDs: 290nm +/− 5 nm with UVB light intensity of UVB 0.0377 W/m² (2 h = 271 W/m²) and UVA light intensity of UVA 0.00062 W/m² (Ferdinand-Braun Institut, Leibniz-Institut für Höchstfrequenztechnik, Berlin, Germany; Supplemental Materials Figure S2) instead of the broad-banded UV light treatment. The controls were further grown under broad-banded UV light. On the 14th day, 24 h after the last narrow-banded UVB light treatment, the plants were harvested. Kale and pea leaves were harvested as five biological replicates. The entire leaves were frozen in liquid nitrogen, lyophilized, and then ground to a fine powder to maximize homogeneity for further analysis. The remaining fresh plant material was used for the breadmaking experiments.

### 2.3. Breadmaking Experiments

Breadmaking experiments were conducted according to Klopsch et al. [22] with slight modifications. Initially, two different doughs were prepared. The first dough basis was for a plain mixed-wheat bread consisting of 41% wheat flour (Type 550, Baeko Hansa eG, Germany, Hamburg), 30% water, 8.5% rye flour (Type 1150, Baeko Hansa eG, Germany, Hamburg), 18% sour dough (rye flour + starter culture; Reinzucht-Sauerteig, Ernst Boecker GmbH and Co. KG, Germany, Minden), 1.5% salt (European Salt Company GmbH and Co. KG, Hannover, Germany), and 1.2% yeast (Uniferm GmbH and Co. KG, Werne, Germany). This served as the control, i.e., non-enriched bread. For the second dough, the plain mixed-wheat bread was modified by adding 4.1% red lentil flour (Müllers Mühle GmbH, Gelsenkirchen, Germany), thus supplementing 8.1 g protein per 500 g bread. The control dough and the lentil dough were then enriched with 10.7% fresh plant material to produce eight different types of bread: (I) broad-banded UV kale-enriched bread, (II) broad-banded UV kale-enriched bread with lentil flour, (III) narrow-banded UVB kale-enriched bread, (IV) narrow-banded UVB kale-enriched bread with lentil flour, (V) broad-banded UV pea-enriched bread, (VI) broad-banded UV pea-enriched bread with lentil flour, (VII) narrow-banded UVB kale-enriched bread, and (VIII) narrow-banded UVB pea-enriched bread with lentil flour. After rising the dough for 40–50 min in a fermentation chamber, the breads were baked for 40 min at 230 °C. After cooling, the breads were cut in small cubes (2 cm × 2 cm), frozen at −40 °C, lyophilized, and ground. Both crumb and crust were used for analyses.

### 2.4. Antioxidant Activity

Antioxidant activity (AA) can differ depending on the assay used. For example, the DPPH assay and the ferric ion reducing antioxidant power (FRAP) assay were shown to be less sensitive for kaempferol glycosides [26]. Thus, to prevent inaccurate interpretation of radical scavenging activity, the Trolox^®^ equivalent antioxidant capacity (TEAC) assay was used in addition to the DPPH assay for comparison purposes. Lyophilized, ground samples (plant material: 10 mg; bread: 100 mg) were extracted using 60% aqueous methanol and a thermomixer at 1400 rpm, 20 °C, and 40 min. Samples were centrifuged at 19,000× *g* for 10 min at 20 °C, and the supernatants were collected in individual 2 mL Eppendorf tubes. This extraction procedure was repeated twice. The supernatants were combined and evaporated to dryness in a rotary evaporator. The residues were suspended in 200 μL of 10% aqueous methanol and transferred to Spin-X tubes with a 0.22 μm cellulose acetate membrane (Corning^®^ Costar^®^ Spin-X^®^, Sigma Aldrich Chemical Co., St. Louis, MI, USA). Samples were centrifuged at 850× *g* for 5 min at 20 °C and transferred to 1 mL vials. Plant extracts were then diluted 1:5 with 10% aqueous methanol and bread extracts were diluted 1:2 with 10% aqueous methanol.

#### 2.4.1. Trolox Equivalent Antioxidant Activity (TEAC) Assay

The TEAC assay, as described by Fiol et al. [27], was performed with the following modifications: An ABTS• working solution (0.06 mM) with 9.6 mg ABTS•, 1.66 mg potassium persulfate, 25 mL milliQ water, and 20 mL methanol was prepared. Each 100 µl of diluted plant extracts or diluted bread extracts were transferred to a 4 mL cuvette and mixed with 2400 µl ABTS• working solution. After 6 min, the extinction was measured with a spectrophotometer at 734 nm (UVi Line 9100; Schott Instruments GmbH, Mainz, Germany). Results were evaluated using a Trolox^®^ calibration curve (2.5 mM) and expressed as mM Trolox^®^ mg^−1^ DM. Each sample was analyzed in three separate replicates.

#### 2.4.2. 2,2-Diphenyl-1-Picrylhydrazyl (DPPH) Assay

The DPPH assay as described by Brand-Williams [28] was performed with the following modifications: A DPPH working solution (0.06 mM) was prepared with 5.91 mg DPPH and 25 mL methanol. Each 100 µL of diluted plant extracts or diluted bread extracts were transferred to a 4 mL cuvette and mixed with 2400 µL DPPH working solution. After 30 min, the extinction was measured with a spectrophotometer at 515 nm (UVi Line 9100; Schott Instruments GmbH, Mainz, Germany). Results were evaluated via the Trolox^®^ calibration curve (2.5 mM) and expressed as mM Trolox^®^ mg^−1^ DM. Each sample was analyzed in three separate replicates. 

### 2.5. Analysis of Carotenoids and Chlorophylls by Ultrahigh Perfomance Liquid Chromatography-Diode Array Detection-Time of Flight-Mass Spectrometry (UHPLC-DAD-ToF-MS)

Extraction of carotenoids and chlorophyll metabolites was conducted according to Klopsch et al. [22]. Analysis of carotenoids and chlorophyll metabolites was performed immediately after extraction using an Agilent Technologies 1290 Infinity II UHPLC (Agilent Technologies Sales and Services GmbH and Co. KG, Waldbronn, Germany) according to Klopsch et al. [22]. The results were expressed in µg g^−1^ FW as mean ± standard deviation (SD) of five biological repetitions of each non-processed plant material and two biological repetitions (each three technical replicates thereof) of the control bread and each plant-enriched bread (‘vegetable bread’). The experiments (plant growth, UVB exposure, breadmaking) were carried out with two replicates for each. To evaluate the extent to which the bread matrix influences the recovery rate of the metabolites, the recovery rates of the carotenoids in the bread matrix were determined. Known amounts of standard substances (β-carotene and lutein) were added to the ‘control bread’, the ‘lentil bread’ (without plant material), and to methanol/tetrahydrofuran (1:1) in three separate replicates each. ‘Control bread’, ‘lentil bread’, and methanol/tetrahydrofuran with the added standards were extracted and analyzed as described above. The recovery rate for both compounds was 89%–94%. 

### 2.6. Analysis of Flavonoid Glycosides and Hydroxycinnamic Acid Derivatives by High Perfomance Liquid Chromatography-Diode Array Detection-Electrospray Ionisation-Mass Spectrometry (HPLC-DAD-ESI-MS^N^)

For the analysis of flavonoid glycosides and hydroxycinnamic acid derivatives, samples were extracted according to Klopsch et al. [22]. Analysis of flavonoid glycosides and hydroxycinnamic acid derivatives was performed with an Agilent HPLC series 1100 (Agilent Technologies Deutschland GmbH, Waldbronn, Germany) according to Klopsch et al. [22]. The results were expressed in mg g^−1^ FW as mean ± SD of five biological repetitions of the non-processed plant material and two biological repetitions (each three technical replicates thereof) of the control bread, the lentil bread, or each plant-enriched bread. The entire experiments (plant growth, UVB, breadmaking) were carried out in two replicates each. To evaluate the extent to which the bread matrix influences the recovery of the phenolics, the recovery rates of sinapic acid, kaempferol-3-*O*-glucoside, and quercetin-3-*O*-glucoside in the bread matrix were determined. Known amounts of the standards sinapic acid, kaempferol-3-*O*-glucoside, and quercetin-3-*O*-glucoside were added to the ‘control bread’ and the ‘lentil bread’ (without plant material) and to methanol (60%) each in three replicates. ‘Control bread’, ‘lentil bread’, and methanol with the added standards were extracted and analyzed as described above. The recovery rate for these compounds was 84%–90%. 

### 2.7. Analysis of Protein Content by the Kjeldahl Method

Crude protein contents of the control bread, lentil flour, and the control bread with lentil flour were determined using the Kjeldahl method [29]. A Speed Digester K-425 and a distillation device K-355 (both Büchi Labortechnik GmbH, Flawil, Switzerland) were used for the analysis. Samples were weighed (1–2 g) and transferred into a Kjeldahl digestion flask containing a Kjeldahl catalyst pellet (TCT, Carl Roth, Germany) and 25 mL concentrated sulfuric acid. After 3 h of digestion followed by 30 min of cooling down to room temperature, the digestion flask was transferred to the distillation device. Before the distillation started, 75 mL of water and 75 mL NaOH (mass concentration = 33%) were added to each flask. During distillation, NH_3_ was trapped in a boric acid solution (mass concentration = 4%). Total nitrogen was determined by titration with 0.1 M HCl. Finally, to calculate the crude protein content in each sample, the total nitrogen content was multiplied by a specific factor of 6.2 [29].

### 2.8. Data Handling and Statistical Analysis

Data were expressed as mean ± SD. The statistical analyses were carried out using Statistica 12 for Windows (Version 9.0, Statsoft Inc., Tulsa, OK, USA). Statistical significance was defined for *p* ≤ 0.05 (95% confidence level). The differences between the two light treatments (broad-banded UV and narrow-banded UVB) and the bread samples were tested by the Student’s *t*-Test. Normal distribution of data in the different samples was tested. Data for the AA was statistically analyzed by a three-factorial ANOVA. Fisher’s F-test was performed to assess the main effects of the pre-harvest, narrow-banded UVB treatment, plant species, lentil flour, and possible polyphenol-protein interactions. This was then followed by a comparison of the factor levels using Tukey’s honestly significant difference (HSD) test.

## 3. Results and Discussion

To study the impact of pre-harvest, narrow-banded UVB treatment on SPMs, kale and pea plants were grown temporarily with narrow-banded UVB and as a control under broad-banded UV. These kale or pea leaves were used to enhance a plain mixed-wheat bread and make ‘vegetable breads’, in order to study the different SPM profile stabilities during a breadmaking process. 

### 3.1. Antioxidant Activity of the Breads

**Figure 1:**
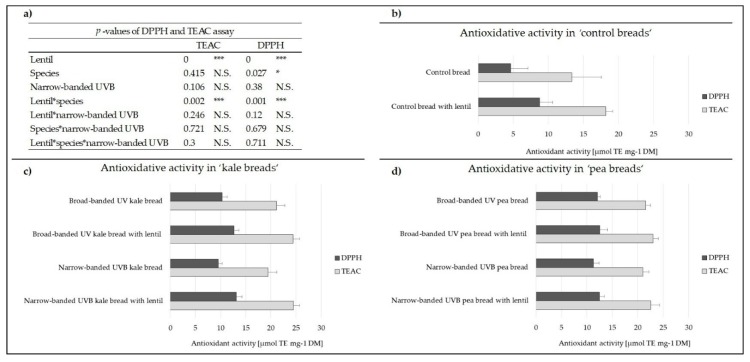
Total antioxidant activity in µmol trolox equivalent (TE) mg^−1^ DM including *p*-values (N.S. = not significant). (**a**) *p*-values, (**b**) control breads, (**c**) ‘kale breads’, and (**d**) ‘pea breads’. * = *p*-value < 0.01 and *** = *p*-value < 0.005.

To evaluate the potential of the ‘vegetable breads’ as Functional Food, it is necessary to assess the AA of the ‘vegetable breads’ compared to the control bread. Further, the presence of proteins can influence the bioavailability of phenolic compounds from the food matrix. Polyphenol–protein interactions are also known to be influenced by temperature, pH, and chemical structure of the substances and their presence could reduce the AA of the phenolic compounds as a result of the covalent attachment to the protein [10,11]. The TEAC and DPPH assays were used to evaluate the effect of the addition of lentil flour as well as non-processed kale or pea plant material on the total AA in the eight different breads. In addition, whether the AA differs in bread enriched with plant material exposed to narrow-banded UVB compared to bread enriched with material from broad-banded UV plants was investigated. The three-factorial ANOVA revealed a significant influence of lentil flour in both assays, a slight effect of the specific species in the DPPH assay, but not in TEAC assay, and no effect of UVB exposure on the overall AA for the different breads (Figure 1). Of note is that all ‘vegetable breads’ showed an increase in the AA compared to the control bread (Figure 1, Supplemental Materials Table S1). 

The AA of ‘vegetable breads’ enriched with narrow-banded UVB plant material did not differ from ‘vegetable breads’ enriched with broad-banded UV plant material (Figure 1, Supplemental Materials Table S1). In ‘kale breads’, the AA increased 2–3-fold as measured by the DPPH assay and 1.5-fold for the TEAC assay. This increase was similar in ‘pea breads’ with a 2.5-fold increase measured by the DPPH assay and a 1.5-fold increase by the TEAC assay (Figure 1, Supplemental Materials Table S1). ‘Kale breads’ and ‘pea breads’ showed a similar AA even though ‘pea breads’ had a higher flavonoid glycoside concentration. This could be explained by the fact that the AA of flavonoid glycosides is highly dependent on their specific structure, e.g., glycosylation and acylation (see Section 3.3.1.) [27]. Lentil flour increased the AA of control bread by 0.4–0.8-fold compared to control bread without lentil flour (Figure 1, Supplemental Materials Table S1), probably because phenolic compounds – and other SPMs – in the lentil flour itself might contribute to its AA [4,8]. In ‘kale breads’ enriched with lentil flour, the AA was 15%–40% higher compared to ‘kale breads’ without lentil flour, and in ‘pea breads’ with lentil flour the AA was 4%–10% higher compared to ‘pea breads’ without lentil flour (Figure 1, Supplemental Materials Table S1). Lentils have been previously shown to possess a moderate level of AA [8]. It is therefore not unreasonable to suggest that they could confer this property, and thus, enhance the overall AA of all of the ‘vegetable breads’. However, the increase of AA of the respective ‘vegetable bread’ and ‘lentil bread’ was not directly proportional and did not sum up exactly, which might have been caused by polyphenol–protein interactions within the matrix [10]. In conclusion, due to their highly increased AA, ‘vegetable breads’ could act as a Functional Food with the addition of lentil flour further increasing this health-promoting property.

### 3.2. Carotenoids and Chlorophylls

#### 3.2.1. Broad-Banded UV Light versus Narrow-Banded UVB Light

**Figure 2:**
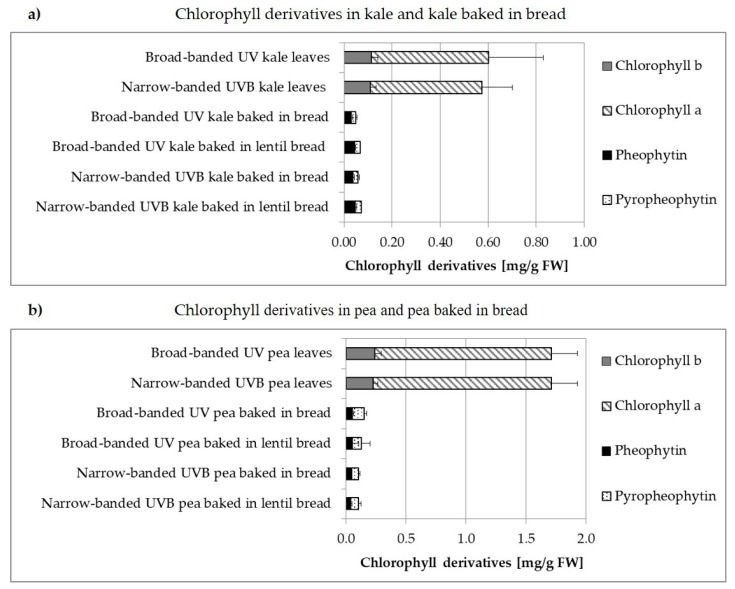
Chlorophyll derivatives in (**a**) kale baked in bread and (**b**) pea baked in bread in mg/g FW. Concentrations of chlorophyll in ‘kale baked in (lentil) bread’ and ‘pea baked in (lentil) bread’ refer to the proportion of plant material, hereby simplifying the comparability of the results of the ‘vegetable breads’ with the results of the corresponding plants.

Carotenoids and chlorophyll metabolites of kale and pea leaves were identified by means of retention time, specific mass (*m/z*), and UV absorption maxima obtained by UHPLC-DAD-ToF-MS (Table S1). In both kale and pea, lutein, β-carotene, neoxanthin, violaxanthin, chlorophyll a, and chlorophyll b were identified (Table 1, Figure 2). Additionally, in kale, α-carotene was also found (Table 1). 

The results for carotenoids in 14-day kale leaves differed from those found in our own recent study [21]. In detail, the carotenoid concentration was generally lower in kale leaves and α-carotene was exclusively found in kale leaves in the present study. The discrepancies could be caused by different growing conditions since in the present study, kale was grown in trays for 14 days without being separated (Supplemental Materials Figure S1), whereas in the previous study, plants were transferred to separate pots after seven days of plant growth [21]. It is therefore conceivable that the plants grown in trays were exposed to a higher stress level resulting from competition for nutrients and space from day eight onwards [30,31,32]. In addition, the plants grown in trays were probably exposed to less light since the leaves from other plants shaded each other. Of note is that shading has been shown to be able to decrease the β-carotene concentration and to increase the α-carotene concentration [33]. In line with these findings, the β-carotene concentration was higher in kale leaves grown in single pots [21] and α-carotene was only found in kale leaves from the present study (Table 1). In kale and peas leaves treated with pre-harvest, narrow-banded UVB, only α-carotene and neoxanthin derivative 2 showed an increase. In pea, only violaxanthin derivative 2 showed an increase resulting from pre-harvest, narrow-banded UVB treatment. Of interest is that also in pak choi (*Brassica rapa* subsp. *chinensis*) and broccoli sprouts (*Brassica oleracea* var. *italic*), changing UV/UVB conditions caused no or only minor changes in carotenoid concentration [12,34]. Other studies showed changes in the concentration of carotenoids and chlorophylls as a result of UVB radiation. For example, in broccoli sprouts, single UVB treatment (280–310 nm, 120 min) caused an increase of lutein and chlorophyll b [13]. In tomato, an increase of carotenoid accumulation after exposure to UVB was also shown [35,36]. The response of carotenoids and chlorophyll metabolism to UVB exposure involves a variety of factors, such as phytohormones and enzymes, and is also highly dependent on plant species, plant tissue, developmental stage, time period, as well as the intensity of the UVB radiation [13,17,30,35]. In the present study, the applied dose of narrow-banded UVB radiation elicited no significant effect on either the carotenoid or chlorophyll concentration in both kale and pea plant material.

#### 3.2.2. Non-Processed versus Processed

Control mixed wheat bread only and mixed wheat bread enriched with lentil flour were further enriched with broad-banded UV kale and pea leaves or narrow-banded UVB kale and pea leaves (‘vegetable breads’). Neither of the two control mixed wheat breads contained carotenoid or chlorophyll derivatives. In the ‘vegetable breads’, lutein was the only carotenoid that could be recovered after breadmaking and was between 1.8%–2.1% and 9%–15.1% in all versions of the ‘kale breads’ and ‘pea breads’ compared to the respective non-processed plant material (Table 1). Our own previous studies on kale and pea also revealed major decreases (>80%) of carotenoids as a result of the breadmaking process with 20% and 6% of lutein being recovered in ‘kale leaf bread’ and ‘pea leaf bread’, respectively [21,22]. The far lower recovery rates of lutein in this study compared to previous ones could be caused by different initial lutein concentrations, possibly due to different growing conditions [30,32], as already discussed (see Section 3.3.1.). In the present study, chlorophyll was not found in any of the ‘vegetable breads’. Heat treatment can lead to the degradation of chlorophyll – a process that is accompanied by pheophytin followed by pyropheophytin formation [37]. Accordingly, pheophytin and pyropheophytin were found in all ‘vegetable breads’. In ‘kale breads’, pheophytin corresponded to 6.3%–8% and pyropheophytin to 3.5%–4.6% of the initial chlorophyll concentration (Figure 2a). These percentages are consistent with our own previous studies on bread enriched with *Brassica* leaves [21]. Interestingly, whereas in ‘kale breads’, the pheophytin concentration exceeded that of pyropheophytin, these concentrations were the other way around in ‘pea breads’ since 2.3%–3.1% pheophytin and 3.6%–5.6% pyropheophytin of the initial chlorophyll concentration were found in the ‘pea breads’ after breadmaking in the present study (Figure 2b). The formation of chlorophyll degradation products was shown to be dependent on several factors, including processing method, temperature, and duration of the thermal treatment [38]. Moreover, whereas blanching and steaming led to a higher pheophytin formation in spinach leaves, microwaving and heat processing produced similar or even higher amounts of pyropheophytin compared to pheophytin [38,39]. Taken together, in the present study, all ‘vegetable breads’ contained lutein and chlorophyll degradation products and could therefore serve as Functional Foods due to the increased number of bioactive compounds. 

### 3.3. Flavonoid Glycosides and Hydroxycinnamic Acid Derivatives

#### 3.3.1. Broad-Banded UV-Light versus Narrow-Banded UVB-Light

Flavonoid glycosides and hydroxycinnamic acid derivatives of kale and pea leaves were tentatively identified by means of retention time and mass spectra obtained by HPLC-DAD-ESI-MS^n^ (Supplemental Materials Tables S2–S4) [21,22]. In kale and pea, flavonoid glycosides based on kaempferol and quercetin and hydroxycinnamic acid derivatives were found (Table 2 and Table 3, Supplemental Materials Tables S2–S4).

In kale, to the best of our knowledge, kaempferol glycosylated with rhamnose, proposed as kaempferol-3-*O*-dirhamnoside-7-*O*-glucoside, was tentatively identified for the first time (Supplemental Materials Figure S2). Kaempferol-3-*O*-dirhamnoside-7-*O*-glucoside was also characterized according to Onkokesung et al. [40] by *m/z* 739 in MS, followed by the loss of one glucoside (M–H–162)¯ in MS^2^ leading to the fragment ion 577 and further in MS^3^, the loss of dirhamnoside (M–H–292)¯ leading to the aglycon kaempferol *m/z* 285 (Supplemental Materials Table S3, Figure S2). This compound was not found in our other recent studies on kale [21]. Besides the newly found kaempferol-3-*O*-dirhamnoside-7-*O*-glucoside, the composition, but not concentrations, of flavonoid glycosides and hydroxycinnamic acid derivatives were similar in kale leaves, as reported by Klopsch et al. [21], compared to the present study. However, the concentration of these metabolites was 5-fold higher in the present study and the ratio also differed. These discrepancies could be caused by different growing conditions since kale plants were grown in trays for 14 days without being separated and probably were exposed to a higher stress level due to competition for nutrients and space. In addition, the leaves shaded each other as the plants were quite close. Of note is that shading often leads to decreased flavonoid accumulation, but in some cases also to increased flavonoid accumulation [4], which may have taken place in the present study. Further, changes in the glycosylation of flavonoid glycosides to adjust to different stress factors is known to occur [41]. Hence, the formation of rhamnoside could be a possible response to abiotic stress. In kale, hydroxycinnamic acid derivatives based on caffeic acid, ferulic acid, hydroxyferulic acid, and sinapic acid, were found (Table 2a) [24]. In pea, only hydroxycinnamic acid derivatives based on coumaric and caffeic acid were found [25]. Thus, the variety and complexity of hydroxycinnamic acid derivatives was higher in kale compared to pea. Moreover, in kale treated with narrow-banded UVB, sinapic acid-glucoside increased and sinapoyl-feruloyl-gentiobiose decreased compared to kale exposed to broad-banded UV only. Accordingly, the biosynthesis of sinapic acid-glucoside compared to sinapoyl-feruloyl-gentiobiose is less complex, and thus, maybe more favorable. Moreover, the conversion of sinapoyl-feruloyl-gentiobiose to sinapic acid-glucoside might also be possible since sinapic acid is thought to play a protective role against UVB [41]. In pea, only coumaroyl-glucoside was increased as a result of pre-harvest, narrow-banded UVB treatment. In the phenylpropanoid pathway, *p*-coumaric acid is the hydroxycinnamic acid which is first formed [42]. Hence, an increase of coumaroyl-glucoside due to exposure to narrow-banded UVB light would be a relatively fast response. The remaining hydroxycinnamic acid derivatives in both kale and pea showed no significant effects resulting from pre-harvest, narrow-banded UVB treatment. Concurrent to the hydroxycinnamic acid derivatives, flavonoid glycosides showed a higher variety in kale compared to pea. Further, the degree of glycosylation was also more complex in kale compared to pea (Table 2b,c and Table 3b,c). The type of glycosylation as well as acylation of flavonoid glycosides can have a decisive influence on the AA of the respective flavonoid glycosides [27,43]. Further, kaempferol-3-*O*-hydroxyferuloyl-sophoroside-7-*O*-glucoside and quercetin-3-*O*-sinapoyl-sophoroside-7-*O*-ᴅ-glucoside, both found in kale but not in pea, were found to possess a high AA according to Fiol et al [27]. In pea, five out of eleven flavonoid glycosides were acylated with coumaroyl. Flavonoid glycosides acylated with coumaroyl showed only minor AA in kale [27]. It was therefore assumed that both species would react differently to narrow-banded UVB treatment. Treatment of kale with narrow-banded UVB radiation led to a decrease of five out of seven kaempferol glycosides with a simultaneous increase of four out of six quercetin glycosides (Table 2b,c). This trend is in line with earlier studies [44] and also underlined by the increase of the quercetin to kaempferol ratio from broad-banded UV kale (0.5) to narrow-banded UVB kale (0.7), which was previously shown to be a general mechanism of plants in response to increased exposure to UVB [45]. This increased ratio could be explained by a conversion of kaempferol glycosides to quercetin glycosides since light-responsive flavonoids with a dihydroxylated B-ring (here quercetin) were shown to have a stronger ability to inhibit reactive oxygen species (ROS) formation or quench ROS activity compared to their corresponding structural counterparts with a monohydroxylated B-ring (here kaempferol) [45]. In summary, the total flavonoid concentration decreased by 11% in narrow-banded UVB kale compared to broad-banded UV kale. This finding implies that one part of the synthesized quercetin glycosides was already used for ROS quenching [45]. In narrow-banded UVB-exposed pea, only kaempferol-3-*O*-sophorotriose derivative 1 and quercetin-3-*O*-sophorotriose derivative 1 increased and kaempferol-3-*O*-sophorotriose derivative 2 decreased, possibly by a conversion to one of the two increasing derivatives (Table 3b,c) [45]. Moreover, the quercetin to kaempferol ratio slightly decreased in narrow-banded UVB pea (3.8) compared to broad-banded UV pea (4.1). However, the total flavonoid concentration in narrow-banded UVB pea was similar compared to broad-banded UV pea. The impact of UVB on the flavonoid accumulation is known to be dependent on duration, intensity, and wavelength of the UV radiation [4,17]. The lower impact of narrow-banded UVB light treatment on pea compared to kale implies that such effects are also dependent on the plant species as well as the chemical structures of the flavonoids [17,46].

#### 3.3.2. Non-Processed versus Processed

Control mixed wheat bread only and mixed wheat bread enriched with lentil flour were further enriched with broad-banded UV kale and pea leaves or narrow-banded UVB kale and pea leaves (‘vegetable breads’). In the control bread, no flavonoid glycosides or hydroxycinnamic acids derivatives were found. In the control breads with lentil flour, the phenolic compounds found showed neither co-elution nor interaction with phenolic compounds found in kale and pea. Enrichment with lentil flour was carried out with the explicit aim of increasing the protein content of the bread. Thus, the very low amounts of phenolic compounds found in the breads with lentil flour will not be discussed further in this study. In ‘kale breads’, 19%–20% of the hydroxycinnamic acid derivatives and 16%–19.8% of the flavonoid glycosides found in non-processed kale leaves were recovered. Thereby, pre-harvest, narrow-banded UVB treatment has an impact on the stability of single compounds (Table 2). In ‘narrow-banded UVB kale bread’, three out of seven kaempferol glycosides showed a higher recovery rate after breadmaking compared to ‘broad-banded UV kale bread’. On the other hand, three other kaempferol glycosides and three out of six quercetin glycosides showed a lower recovery rate after breadmaking in ‘narrow-banded UVB kale bread’ compared to ‘broad-banded UV kale bread’ (Table 2b,c). These effects were independent of whether the dough was enriched with lentil flour or not. In fact, no differences in the recovery of hydroxycinnamic acid derivatives and flavonoid glycosides in ‘kale breads’ made with or without lentil flour were found (Table 2a–c), and thus, presumably, they were caused by the pre-harvest, narrow-banded UVB treatment. Narrow-banded UVB kale leaves were exposed to two stress situations: (1) the pre-harvest narrow-banded UVB treatment and (2) the breadmaking process. Agati, Azzarello, Pollastri, and Tattini [45] proposed that the activity and stability of flavonoids is also dependent on their subcellular distribution. Hence, the altered stability of these compounds during breadmaking could be caused by narrow-banded UVB-induced changes in inter- and intra-cellular distributions of the respective flavonoid glycosides and the alteration of their integration in different cells/cell compartments [45,47]. Contrary to the recovery rates in ‘kale breads’ in the present study, our own recent study found a 2–3-fold increase of hydroxycinnamic acid derivatives and a 84% recovery rate for flavonoid glycosides in ‘kale leaf breads’ [21]. However, in the present study, the concentration of flavonoid glycosides and hydroxycinnamic acid derivatives was 5-fold higher compared to Klopsch et al. [21] and the ratio also differed. This increase was probably caused by different growing conditions as already previously discussed (see Section 3.3.1.). Although the initial concentration of flavonoid glycosides and hydroxycinnamic acid derivatives in kale leaves differs in Klopsch et al. [21] and in the present study, in the end, the total concentrations in the ‘kale breads’ was similar for both experiments. Flavonoids occur in a variety of cell compartments and can interact—both physically and chemically—with the respective membranes [45]. The way in which flavonoids are bound as well as the (transport) mechanisms and velocity by which they are made available when stress occurs also differ [4,45]. Of note is that dependent on their chemical structure and localization, they can be made faster available for plant protection during stress [4,45]. Thus, the similar absolute concentration of phenolic compounds in the ‘kale breads’ from the present and the recent study [21] might therefore be explained by the ability of the plant to stabilize and retain a threshold of flavonoid glycosides and hydroxycinnamic acid derivatives during stress. As mentioned earlier, flavonoid glycosides found in pea were less complex regarding glycosylation compared to flavonoid glycosides in kale. It can be assumed that the stability of flavonoid glycosides during thermal processing also depends on their specific chemical structure [48,49]. In fact, flavonoid glycosides as well as hydroxycinnamic acid derivatives found in pea showed a higher stability during breadmaking compared to those found in kale. In ‘pea breads’, 31%–37% of the hydroxycinnamic acid derivatives and 81%–99% of the flavonoid glycosides found in the respective non-processed pea material were recovered after breadmaking. The high stability of flavonoids is in accordance with our recent study on breadmaking with fresh pea tissue [22]. ‘Narrow-banded UVB pea bread’ made without lentil flour showed a lower recovery of hydroxycinnamic acid derivatives and flavonoid glycosides compared to ‘broad-banded UV pea bread’ made without lentil flour (Table 3b,c). Presumably, this lower recovery rate was also caused by the two successive stress situations, namely pre-harvest, narrow-banded UVB treatment and the breadmaking process, as already discussed for kale [45,47]. In contrast, ‘narrow-banded UVB pea bread’ made with lentil flour showed an increased recovery of total kaempferol and total quercetin glycosides compared to ‘narrow-banded UVB pea bread’ made without lentil flour (Table 3b,c). Hence, the protein-rich lentil flour seems to have a stabilizing effect in breads enriched with narrow-banded UVB, but not in breads made with broad-banded UV pea material. Since polyphenol–protein interactions depend on the chemical structure of the phenolic compounds [10,11], it is not surprising that this stabilizing effect is restricted to ‘pea breads’. The restriction of this effect to ‘narrow-banded UVB pea bread’ could be explained by a higher susceptibility of narrow-banded UVB pea to polyphenol–protein interactions caused by narrow-banded UVB-induced changes of the integration of flavonoid glycosides into different cell compartments or their inter- and intra-cellular distribution [45]. Consistent with earlier findings, the impact of thermal processing on kaempferol and quercetin glycosides varied depending on the glycosylation status, and pattern (Table 3b,c) [22,48]. Hence, changes in the concentration of flavonoid glycosides due to breadmaking were structure-specific. For example, a slight decrease of acylated and glycosylated quercetin derivatives in favor of a slight increase of glycosylated quercetin derivatives, possibly due to deacylation, was found [27,49]. Thus, to better control the impact of thermal processing on phenolic compounds, various intrinsic (plant species, plant tissue, chemical composition of respective substance) and extrinsic (temperature, duration, kind of heat treatment) factors have to be considered. 

## 4. Conclusions

Carotenoids, chlorophylls, and hydroxycinnamic acid derivatives were negligibly affected by pre-harvest, narrow-banded UVB treatment. In kale, kaempferol glycosides were reduced in favor of an increase in quercetin glycosides. In pea, the impact of pre-harvest, narrow-banded UVB treatment was minimal. In the present study, we found that breadmaking with kale or pea leaves led to species- and structure-specific losses of flavonoid glycosides and hydroxycinnamic acid derivatives. Further, the majority of carotenoids and chlorophylls were depleted, and this was accompanied by the formation of pheophytin and pyropheophytin from chlorophylls in all ‘vegetable breads’. Moreover, for the first time, the effect of the addition of lentil flour to bread enriched with fresh vegetables was investigated. The lentil flour was found to not significantly increase the protein content of the ‘vegetable breads’. It did, however, increase their AA. The effects of pre-harvest, narrow-banded UVB, followed by breadmaking and the addition of lentil flour, was also found to be dependent on the plant species and the chemical structure of the respective substance. In addition, exposure of the plant to one stress factor (exposure to narrow-banded UVB) may affect its response to a second, subsequent stress factor (breadmaking). Dependent on the plant species, pre-harvest (narrow-banded UVB), and post-harvest factors (lentil flour) could have stabilizing effects on the desired health-promoting bioactive compounds during breadmaking. All ‘vegetable breads’ showed an enhanced amount of bioactive flavonoid glycosides and hydroxycinnamic acid derivatives, and hence, fulfill the requirements for a Functional Food. Thus, the results obtained in the present study provide a basis for further investigations on the effect of thermal processing on SPMs in UVB-treated leafy vegetables in order to optimize the SPM profile in processed vegetable-enriched products.

## Figures and Tables

**Table 1:** Influence of narrow-banded UVB (ultraviolet B) and breadmaking on carotenoids in (**a**) kale and (**b**) pea leaves.

(a)		Kale Leaves			Kale Bread	Kale Bread with Lentil
		mg/g FW in Plant Tissue			Recovery in Kale Bread (%)	
Lutein	broad-banded UV	0.034 ^A^	±	0.009	1.8 ^a^	2 ^a^
	narrow-banded UVB	0.028 ^A^	±	0.008	1.9 ^a^	2.1 ^a^
α-Carotene	broad-banded UV	0.001 ^A^	±	0.001	N.D.	N.D.
	narrow-banded UVB	0.002 ^B^	±	0.001		
ß-Carotene	broad-banded UV	0.011 ^A^	±	0.004	N.D.	N.D.
	narrow-banded UVB	0.009 ^A^	±	0.003		
Neoxanthin derivative 1	broad-banded UV	0.01 ^A^	±	0.003	N.D.	N.D.
	narrow-banded UVB	0.009 ^A^	±	0.003		
Neoxanthin derivative 2	broad-banded UV	0.004 ^A^	±	0.001	N.D.	N.D.
	narrow-banded UVB	0.005 ^B^	±	0.001		
Violaxanthin	broad-banded UV	0.001 ^A^	±	0.001	N.D.	N.D.
	narrow-banded UVB	0.001 ^A^	±	0.001		
Total carotenoids	broad-banded UV	0.06 ^A^	±	0.019	1.2 ^a^	1.36 ^a^
	narrow-banded UVB	0.055 ^A^	±	0.017	1 ^a^	1.1 ^a^
**(b)**		**Pea Leaves**			**Pea Bread**	**Pea Bread with Lentil**
		**mg/g FW in Plant Tissue**			**Recovery in Pea Bread (%)**	
Lutein	broad-banded UV	0.102 ^A^	±	0.023	15.1 ^a^	11 ^a^
	narrow-banded UVB	0.106 ^A^	±	0.020	9 ^b^	11.3 ^b^
ß-Carotene	broad-banded UV	0.052 ^A^	±	0.008	N.D.	N.D.
	narrow-banded UVB	0.052 ^A^	±	0.009		
Neoxanthin derivative 1	broad-banded UV	0.023 ^A^	±	0.006	N.D.	N.D.
	narrow-banded UVB	0.021 ^A^	±	0.005		
Neoxanthin derivative 2	broad-banded UV	0.003 ^A^	±	0.001	N.D.	N.D.
	narrow-banded UVB	0.003 ^A^	±	0.001		
Violaxanthin derivative 1	broad-banded UV	0.037 ^A^	±	0.015	N.D.	N.D.
	narrow-banded UVB	0.038 ^A^	±	0.012		
Violaxanthin derivative 2	broad-banded UV	0.005 ^A^	±	0.001	N.D.	N.D.
	narrow-banded UVB	0.006 ^A^	±	0.001		
Total carotenoids	broad-banded UV	0.223 ^A^	±	0.053	7 ^a^	6.9 ^a^
	narrow-banded UVB	0.226 ^A^	±	0.047	4.2 ^a^	5.3 ^b^

Results are presented as means ± standard deviation (SD) (*n*=5) in mg g^−1^ fresh weight. N.D. = Not detected. Student’s *t*-test was conducted by Statistica at a significance level of *p* ≤ 0.05. Capital letters label significant differences between the non-processed plants grown only under broad-banded UV and non-processed plants treated with narrow-banded UVB. Small letters label significant differences in the recovery of carotenoid and chlorophyll metabolites after breadmaking in a ‘broad-banded UV kale bread’ and ‘narrow-banded UVB kale bread’ as well as ‘broad-banded UV kale lentil bread’ and ‘narrow-banded UVB kale lentil bread’ and b ‘broad-banded UV pea bread’ and ‘narrow-banded UVB pea bread’ as well as ‘broad-banded UV pea lentil bread’ and ‘narrow-banded UVB pea lentil bread’.

**Table 2:** Influence of narrow-banded UVB and breadmaking on (**a**) Hydroxycinnamic acid derivatives, (**b**) Kaempferol glycosides, and (**c**) Quercetin glycosides, in kale leaves. Abbreviations: caf: caffeoyl; cou: coumaroyl; fer: feruloyl; glc: glucoside; HCA: hydroxycinnamic acids; hfer: hydroxyferuloyl; k: kaempferol; q: quercetin; rha: rhamnoside; sin: sinapoyl; soph: sophoroside; sophtr: sophorotrioside.

(a) Hydroxycinnamic Acid Derivatives		Kale			Kale Bread	Kale Bread with Lentil
		mg/g FW in Plant Tissue			Recovery in Kale Bread (%)	
3-Caffeoylquinic acid	broad-banded UV	0.309 ^A^	±	0.065	14 ^a^	14 ^a^
	narrow-banded UVB	0.259 ^A^	±	0.044	15.7 ^b^	14.5 ^b^
Caffeoylglucoside	broad-banded UV	0.078 ^A^	±	0.020	N.D.	N.D.
	narrow-banded UVB	0.072 ^A^	±	0.009		
Sinapic acid-glucoside	broad-banded UV	0.185 ^A^	±	0.037	21 ^a^	21.2 ^a^
	narrow-banded UVB	0.239 ^B^	±	0.026	15.7 ^b^	16.8 ^b^
Sinapoyl-feruloylgentiobiose	broad-banded UV	0.257 ^A^	±	0.049	15.1 ^a^	15.7 ^a^
	narrow-banded UVB	0.204 ^B^	±	0.034	18.3 ^b^	19 ^b^
Disinapoyl-feruloylgentiobiose	broad-banded UV	0.095 ^A^	±	0.018	33.5 ^a^	33.8 ^a^
	narrow-banded UVB	0.092 ^A^	±	0.012	34.6 ^b^	34.7 ^a^
Sinapoyl-hydroxyferuloylgentiobiose	broad-banded UV	0.089 ^A^	±	0.018	38.5 ^a^	38.5 ^a^
	narrow-banded UVB	0.082 ^A^	±	0.015	39.8 ^b^	40.5 ^b^
Disinapoyl-gentiobiose	broad-banded UV	0.268 ^A^	±	0.051	17.2 ^a^	17.8 ^a^
	narrow-banded UVB	0.263 ^A^	±	0.038	17.7 ^a^	19.4 ^a^
Trisinapoyl-gentiobiose	broad-banded UV	0.184 ^A^	±	0.045	26 ^a^	25.6 ^a^
	narrow-banded UVB	0.203 ^A^	±	0.032	23.6 ^b^	26.2 ^a^
Hydroxycinnamic acid derivatives	broad-banded UV	1.465 ^A^	±	0.302	19.2 ^a^	19.4 ^a^
	narrow-banded UVB	1.413 ^A^	±	0.211	19.4 ^a^	20.2 ^a^
**(b) Kaempferol Glycosides**		**Kale**			**Kale Bread**	**Kale Bread with Lentil**
		**mg/g FW in Plant Tissue**			**Recovery in Kale Bread (%)**	
K-3-*O*-dirha-7-*O*-rha	broad-banded UV	0.153 ^A^	±	0.024	39.9 ^a^	37.6 ^a^
	narrow-banded UVB	0.128 ^B^	±	0.026	48.8 ^b^	47.5 ^b^
K-3-*O*-soph-7-*O*-ᴅ-glc	broad-banded UV	0.163 ^A^	±	0.052	10.1 ^a^	10.7 ^a^
	narrow-banded UVB	0.124 ^A^	±	0.040	6.1 ^b^	7 ^b^
K-3-*O*-cou-soph-7-*O*-ᴅ-glc	broad-banded UV	0.08 ^A^	±	0.023	19.1 ^a^	20.2 ^a^
	narrow-banded UVB	0.035 ^B^	±	0.011	33.6 ^b^	31.8 ^b^
K-3-*O*-caf-soph-7-*O*-ᴅ-glc	broad-banded UV	0.529 ^A^	±	0.141	17.1 ^a^	16.8 ^a^
	narrow-banded UVB	0.388 ^B^	±	0.129	17.3 ^a^	17.1 ^a^
K-3-*O*-fer-soph-7-*O*-glc	broad-banded UV	0.17 ^A^	±	0.045	16.4 ^a^	17.9 ^a^
	narrow-banded UVB	0.1 ^B^	±	0.031	20.4 ^b^	21.7 ^b^
K-3-*O*-hfer-soph-7-*O*-glc	broad-banded UV	1.204 ^A^	±	0.302	11.4 ^a^	11.8 ^a^
	narrow-banded UVB	0.936 ^B^	±	0.192	9.2 ^b^	9.5 ^b^
K-3-*O*-sin-soph-7-*O*-glc	broad-banded UV	0.26 ^A^	±	0.091	19.9 ^a^	21.3 ^a^
	narrow-banded UVB	0.235 ^A^	±	0.076	13.6 ^b^	15.4 ^b^
Kaempferol glycosides	broad-banded UV	2.56 ^A^	±	0.678	15.6 ^a^	16 ^a^
	narrow-banded UVB	1.945 ^B^	±	0.504	14.8 ^a^	15.1 ^a^
**(c) Quercetin glycosides**		**Kale**			**Kale Bread**	**Kale Bread with Lentil**
		**mg/g FW in Plant Tissue**			**Recovery in Kale Bread (%)**	
Q-3-*O*-triglc	broad-banded UV	0.495 ^A^	±	0.69	10.3 ^a^	10.2 ^a^
	narrow-banded UVB	0.624 ^B^	±	0.109	7.2 ^b^	8.5 ^b^
Q-3,7,4´-*O*-ᴅ-triglc	broad-banded UV	0.145 ^A^	±	0.034	23.6 ^a^	25.5 ^a^
	narrow-banded UVB	0.132 ^A^	±	0.040	23.1 ^a^	22.6 ^b^
Q-3-*O*-caf-soph-7-*O*-glc	broad-banded UV	0.15 ^A^	±	0.047	40.2 ^a^	35.1 ^a^
	narrow-banded UVB	0.195 ^B^	±	0.047	15.5 ^b^	16.1 ^b^
Q-3-*O*-fer-soph-7-*O*-ᴅ-glc	broad-banded UV	0.094 ^A^	±	0.024	N.D.	N.D.
	narrow-banded UVB	0.109 ^A^	±	0.036		
Q-3-*O*-hfer-sophtr-7-*O*-glc	broad-banded UV	0.188 ^A^	±	0.040	75.6 ^a^	77.2 ^a^
	narrow-banded UVB	0.176 ^B^	±	0.041	63.1 ^b^	72 ^a^
Q-3-*O*-sin-soph-7-*O*-ᴅ-glc	broad-banded UV	0.131 ^A^	±	0.044	38.2 ^a^	38.1 ^a^
	narrow-banded UVB	0.175 ^B^	±	0.091	18.7 ^b^	21.4 ^b^
Quercetin glycosides	broad-banded UV	1.203 ^A^	±	0.305	28.1 ^a^	27.9 ^a^
	narrow-banded UVB	1.41 ^A^	±	0.363	17.7 ^a^	19.7 ^b^

Results are presented as means ± SD (*n* = 5) in µg g^−1^ fresh weight. N.D. = Not detected. Student’s *t*-test was conducted by Statistica at a significance level of *p* ≤ 0.05. Capital letters label significant differences between the non-processed kale leaves grown only under broad-banded UV and non-processed kale leaves treated with narrow-banded UVB. Small letters label significant differences in the recovery of phenolic compounds after breadmaking in ‘broad-banded UV kale bread’ and ‘narrow-banded UVB kale bread’ as well as ‘broad-banded UV kale lentil bread’ and ‘narrow-banded UVB lentil kale bread’.

**Table 3:** Influence of narrow-banded UVB and breadmaking on (**a**) Hydroxycinnamic acid glycosides, (**b**) Kaempferol glycosides, and (**c**) Quercetin glycosides, in pea leaves. Abbreviations: caf: caffeoyl; cou: coumaroyl; glc: glucoside; HCA: hydroxycinnamic acids; k: kaempferol; q: quercetin; sin: sinapoyl; soph: sophoroside; sophtr: sophorotrioside.

(a) Hydroxycinnamic Acid Glycosides		Pea						Pea Bread	Pea Bread with Lentil
		mg/g FW in Plant Tissue						Recovery in Pea Bread (%)	
Coumaroyl-glucoside	broad-banded UV	0.022 ^A^	±			0.008		63.5 ^a^	84.5 ^a^
	narrow-banded UVB	0.016 ^B^	±			0.004		47.45 ^b^	79.1 ^a^
Caffeoyl-glucoside 1	broad-banded UV	0.006 ^A^	±			0.001		N.D.	N.D.
	narrow-banded UVB	0.006 ^A^	±			0.001			
Caffeoyl-glucoside 2	broad-banded UV	0.007 ^A^	±			0.001		N.D.	N.D.
	narrow-banded UVB	0.007 ^A^	±			0.002			
Caffeoyl-glucoside 3	broad-banded UV	0.006 ^A^	±			0.001		N.D.	N.D.
	narrow-banded UVB	0.005 ^A^	±			0.001			
Hydroxycinnamic acid derivatives	broad-banded UV	0.041 ^A^	±			0.012		34.3 ^a^	39.7 ^a^
	narrow-banded UVB	0.034 ^A^	±			0.005		25.7 ^a^	37.2 ^b^
**(b) Kaempferol Glycosides**		**Pea**						**Pea Bread**	**Pea Bread with Lentil**
		**mg/g FW in Plant Tissue**						**Recovery in Pea Bread (%)**	
K-3-*O*-sophtr 1	broad-banded UV	0.086 ^A^	±		0.013			111 ^a^	94.4 ^a^
	narrow-banded UVB	0.098 ^B^	±		0.01			109.7 ^a^	107.6 ^b^
K-3-*O*-sophtr 2	broad-banded UV	0.024 ^A^	±		0.014			N.D.	N.D.
	narrow-banded UVB	0.011 ^B^	±		0.005				
K-3-*O*-cou-sophtr 1	broad-banded UV	0.681 ^A^	±		0.108			92.4 ^a^	77.5 ^a^
	narrow-banded UVB	0.742 ^A^	±		0.114			82.1 ^a^	85.5 ^b^
K-3-*O*-cou-sophtr 2	broad-banded UV	0.043 ^A^	±		0.008			61.5 ^a^	66.5 ^a^
	narrow-banded UVB	0.039 ^A^	±		0.004			52.3 ^a^	71.2 ^a^
K-3-*O*-sin-sophtr	broad-banded UV	0.089 ^A^	±		0.019			99.9 ^a^	89.4 ^a^
	narrow-banded UVB	0.093 ^A^	±		0.013			86.8 ^b^	89.2 ^a^
Kaempferol glycosides	broad-banded UV	0.922 ^A^	±		0.128			91.1 ^a^	79 ^a^
	narrow-banded UVB	0.984 ^A^	±		0.13			81.6 ^b^	86.5 ^a^
**(c) Quercetin Glycosides**		**Pea**						**Pea Bread**	**Pea Bread with Lentil**
		**mg/g FW in Plant Tissue**						**Recovery in Pea Bread (%)**	
Q-3-*O*-sophtr 1	broad-banded UV	0.354 ^A^		±			0.061	111.1 ^a^	93.8 ^a^
	narrow-banded UVB	0.409 ^B^		±			0.02	106.6 ^a^	112 ^a^
Q-3-*O*-sophtr 2	broad-banded UV	0.065 ^A^		±			0.028	178.9 ^a^	163.1 ^a^
	narrow-banded UVB	0.07 ^A^		±			0.018	168.5 ^a^	187.6 ^b^
Q-3-*O*-cou-sophtr 1	broad-banded UV	3.007 ^A^		±			0.28	100.3 ^a^	92.4 ^a^
	narrow-banded UVB	2.896 ^A^		±			0.208	75.9 ^b^	91.9 ^a^
Q-3-*O*-cou-sophtr 2	broad-banded UV	0.114 ^A^		±			0.016	109.6 ^a^	91.6 ^a^
	narrow-banded UVB	0.122 ^A^		±			0.041	99.1 ^a^	103.6 ^a^
Q-3-*O*-cou-sophtr 3	broad-banded UV	0.169 ^A^		±			0.045	68.9 ^a^	78.9 ^a^
	narrow-banded UVB	0.137 ^A^		±			0.019	50.1 ^b^	72.3 ^a^
Q-3-*O*-caf-sophtr	broad-banded UV	0.063 ^A^		±			0.017	108.5 ^a^	89 ^a^
	narrow-banded UVB	0.065 ^A^		±			0.011	96.1 ^b^	99.7 ^a^
Quercetin glycosides	broad-banded UV	3.772 ^A^		±			0.362	101.7 ^a^	93.3 ^a^
	narrow-banded UVB	3.697 ^A^		±			0.261	80.3 ^a^	95.8 ^b^

Results are presented as means ± SD (*n* = 5) in µg g^-1^ fresh weight. N.D. = Not detected. Student’s *t*-test was conducted by Statistica at a significance level of *p* ≤ 0.05. Capital letters label significant differences between the non-processed pea leaves grown only under broad-banded UV and non-processed pea leaves treated with narrow-banded UVB. Small letters label significant differences in the recovery of phenolic compounds after breadmaking in ‘broad-banded UV pea bread’ and ‘narrow-banded UVB pea bread’ as well as ‘broad-banded UV pea lentil bread’ and ‘narrow-banded UVB pea lentil bread’.

## Supplementary Materials

The following are available online at https://www.mdpi.com/2304-8158/8/10/427/s1, Table S1: Spectrometric data to identify carotenoids and chlorophyll metabolites in kale and pea leaves measured by UHPLC-DAD-ToF-MS, Table S2: Fragmentation patterns of hydroxycinnamic acid derivatives in kale and pea leaves measured by HPLC-DAD-ESI-MS^n^, Table S3: Fragmentation patterns of kaempferol glycosides in kale and pea leaves measured by HPLC-DAD-ESI-MS^n^, Table S4: Fragmentation patterns of quercetin glycosides in kale and pea leaves measured by HPLC-DAD-ESI-MS^n^, Figure S1: Picture of plants grown in trays without being separated. Left: Kale; Right: Pea, Figure S2: Spectrometric data of UVB LED (µW/cm² nm), Figure S3: Picture of pea-enriched dough before and after baking. a) Raw dough enriched with pea leaves. b) Left: bread enriched with pea leaves; right: control bread, Figure S4: Retention time and mass spectra of kaempferol-3-*O*-dirhamnoside-7-*O*-glucoside tentatively identified in kale leaves.
